# Expression of CD44v6 and lymphatic vessel density in early gastric cancer tissues and their clinical significance

**DOI:** 10.12669/pjms.35.2.464

**Published:** 2019

**Authors:** Yuting Sun, Xiaowei Yu, Mengdi Li, Zhenhong Zou

**Affiliations:** 1 *Yuting Sun, Queen Mary College of Nanchang University, Nanchang, 330006, Jiangxi, China*; 2 *Xiaowei Yu, First Clinical Medical College, Nanchang University, Nanchang, 330006, Jiangxi, China*; 3 *Mengdi Li, Queen Mary College of Nanchang University, Nanchang, 330006, Jiangxi, China*; 4 *Zhenhong Zou, Department of General Surgery, First Affiliated Hospital of Nanchang University, Nanchang, 330006, Jiangxi, China*

**Keywords:** Gastric cancer, CD44v6, LVD

## Abstract

**Objective:**

To explore the relationships between expression of CD44v6, lymphatic vessel density (LVD) and the clinicopathological parameters of patients.

**Methods:**

One hundred early gastric cancer tissues, 55 high-grade gastric intraepithelial neoplasia (HGIN) tissues, 60 low-grade gastric intraepithelial neoplasia (LGIN) tissues and 60 chronic superficial gastritis tissues were collected and set as gastric cancer group, HGIN group, LGIN group and gastritis group respectively. The expression of CD44v6 and LVD of patients in all the groups were detected using two-step immunohistochemical method to analyze the relationships between the expression of CD44v6 and lymphatic vessel density in early gastric cancer tissues and their relationships with the clinicopathological parameters of patients. The values of LVD in predicting lymph node metastasis in early gastric cancer were evaluated using receiver operating characteristic (ROC) curve.

**Results:**

The positive expression of CD44v6 and LVD in the gastritis group, LGIN group, HGIN group and gastric cancer group gradually increased. The positive expression of CD44v6 and LVD in early gastric cancer tissues were in no correlation with the gender, age, tumor site, maximum diameter, differentiation degree and invasion depth (P>0.05) and in a correlation with lymphatic metastasis and lymphatic vessel invasion (P<0.06). The positive expression of CD44v6 in the early gastric cancer tissues was in a positive correlation with LVD (P<0.05). The analysis of ROC curves suggested that the area under ROC curve of predicting lymphatic metastasis of early gastric cancer with LVD was 0.837 (95% CI: 0.756~0.910), and the cut-off value was 14; the corresponding sensitivity and specificity were 63.6% and 90.2 respectively.

**Conclusion:**

The expression of CD44v6 and LVD in early gastric cancer tissues are in a close correlation with the clinicopathologic features, and joint detection of expression of CD44v6 and LVD can be taken as the indicator of gastric cancer metastasis.

## INTRODUCTION

Gastric cancer is one of the common gastrointestinal malignant cancers, and its incidence ranks the second among different malignant cancer in China.^1^ Although comprehensive treatment which focuses on surgery has achieved a certain effect with the development of new medications, the prognosis of gastric cancer is not ideal. Currently there is no reliable clinical method for predicting gastric cancer. Metastasis of gastric cancer is the leading cause of death of patients.^2^,^3^

Studies have found that CD44 as a kind of transmembrane glycoprotein was the surface receptor of hyaluronidase,^4^,^5^ it participated in the regulation of intracellular environment and had functions of promoting tumor growth, invasion and metastasis, and CD44v6 was one of the major variations of CD44. It was also found that CD44v6 was related to the occurrence and development of malignant cancers such as gastric cancer and colorectal cancer, and its expression could increase at the precancerous stage or early stage of some malignant tumors.^6^,^7^ However, few studies related to the changes of CD44v6 expression in early gastric cancer tissues are available. Lymphatic vessel density (LVD) refers to the number of lymphatic vessels per unit area.^8^ The increase of LVD is related to lymphangiogenesis, while lymphangiogenesis is closely related to lymph node metastasis.^9^ Lymph node metastasis is an important way of metastasis of gastric cancer and also one of the early events of gastric cancer metastasis. Therefore, the increase of LVD may have a certain predictive value for lymph node metastasis of early gastric cancer. In this study, the expressions of CD44v6 and LVD in the early gastric cancer were observed. The relationship between CD44v6 expression, LVD and the clinicopathological parameters of the patients was analyzed to explore the role of them in the occurrence and development of gastric cancer.

## METHODS

### Research data

One hundred gastric cancer patients who were admitted to our hospital between December 2016 and December 2017 and set as the gastric cancer group; all of them were positively diagnosed by histopathological examination. Patients who underwent radical surgery or endoscopic submucosal dissection, did not undergo any anti-cancer treatment before surgery, had cancers on mucosal layer or submucous layer, and had complete clinicopathologic data were included. Patients who had primary cancer in other sites, recurrent gastric cancer or gastric stump cancer were excluded.

There were 68 males and 32 females; they aged (60.6±9.8) years. The maximum diameter of the cancers was (2.18±1.36) cm. As to the position of the cancer, there were 30 cases of gastric cardia or fundus, 28 cases of gastric body and 42 cases of gastric antrum. As regards the depth of invasion, there were 43 cases of mucosal layer and 57 cases of submucosa. As to the degree of histologic differentiation, there were 49 cases of highly and moderately differentiation and 51 cases of lowly differentiation. Thirty-seven patients had lymphatic metastasis, and 63 patients had no lymphatic metastasis; 20 patients had lymphatic vessel invasion and 80 patients had no lymphatic vessel invasion. Of the 120 patients, 60 patients had low-grade intraepithelial neoplasia (LGIN group), and 55 patients had high-grade intraepithelial neoplasia (HGIN group). They did not undergo gastric operation or endoscopic treatment previously and were confirmed having disorder of epithelium structure or heterocyst, including dysplasia and carcinoma in situ, by gastroscopy or postoperative histopathological examination. In the LGIN group, there were 42 males and 18 females; their average age of them was (59.7±11.1) years. In the HGIN group, there were 38 males and 17 females; the average age of them was (60.6±9.3) years. Sixty patients who were confirmed having chronic superficial gastritis by health examination or gastroscopy in our hospital in the same period were selected and set as a gastritis group. Patients who had digestive system discomfort, history of gastric surgery, or had atrophic gastritis, intestinal metaplasia or intraepithelial neoplasia were excluded. In the gastritis group, there were 43 males and 17 females; the average age was (59.4±10.6) years. No significant differences were found in baseline data such as age and sex of different groups. The study was approved by the ethics committee of our hospital and all the patients signed informed consent.

The mouse anti-human D2-40 monoclonal antibody (ready-to-use) (Shanghai Yixin Biotechnology Co., Ltd., China), mouse anti-human CD44v6 monoclonal antibody (concentrated type) (Boiss Company, China), SP-9000 immuno histochemical staining kit and concentrated DAB kit (Beijing Zhongshan Biotechnology Co., Ltd., China) were used.

All specimens were fixed with 4% neutral formaldehyde, embedded by paraffin, and cut into sections which was 4 μm thick. The sections were baked overnight at 65 °C, dewaxed using dimethylbenzene, and gradiently dehydrated using ethanol dehydration. Antigen was repaired by heating the sections along with citric acid buffer. After being cooled to the room temperature, the sections were washed by phosphatic buffer solution (PBS), 5 min each time, for three minutes and incubated under 3% H_2_O_2_ for 8 min to eliminate endogenous peroxidase. After being washed by PBS, five minutes each time, for three minutes the sections were added with CD44v6 monoclonal antibody in a ratio of 1: 250 and mouse anti-human D2-40 monoclonal antibody in a ratio of 1: 25 and incubated at 4 °C overnight. After three times of PBS washing, five minutes each time, fast-type enzyme labeled sheep anti-mouse/rabbit Ig G polymer was added, followed by incubation at room temperature for 15~20 minutes and three times of PBS washing, five minutes each time. Then color development was performed using DAB. After being washed by tap water, redyed using hematoxylin, dehydrated using ethyl alcohol and transparentized using dimethylbenzene, the sections were mounted using neural resin and observed under a microscope.

PBS was taken as the negative control instead of the primary antibody. Two pathologists reviewed the films to determine the results using double blind method. The expression of CD44v6 existence was considered if the positive staining of CD44v6 was located on cytomembrane and distributed linearly or zonally in brownish yellow or brown. Five 400X fields were selected on each section; one hundred cells were counted in each field; the staining intensity of the cells was observed, and the number of positive cells was counted. The results were determined according to the staining intensity and proportion of positive cells. 0 point was given if there was no staining, 1 point if light yellow, 2 points if brownish yellow, and 3 points if sepia. It was scored 0 point if the proportion of positive cells was not higher than 5%, 1 point if > 5% ~ ≤25%, 2 points if > 25% ~ ≤50%, three points if > 50% ~ ≤75%, and four points if > 75%. The expression of CD44v6 was determined positive if the product of the scores of staining intensity and proportion of positive cells was not lower than three. LVD was evaluated using the methods described by Weidner et al.^10^ Lumen which was stained as deep yellow was determined as lymphatic vessels. Two sections were taken from each case; three areas with the highest degree of vascularization which were found in D2-40 immunostaining under low-power field (X40) were carefully examined. The average value of the number of lymphatic vessels which were D2-40 positive under three 200X fields was used for representing LVD.

### Observation indexes

The clinicopathological data of the patients with early gastric cancer were collected, including sex, age, and maximum diameter of cancer, tumor site, and depth of invasion, degree of tissue differentiation, lymph node metastasis and lymphatic infiltration.

### Statistical Analysis

SPSS21.0 was used. Measurement data were represented by mean±standard deviation (SD), and the results were compared by independent sample t test or one-way analysis of variance. Enumeration data were compared using Chi-square test. The relationships between the positive expression of CD44v6, LVD and the clinicopathological parameters of the patients were analyzed. Spearman rank correlation analysis was used to analyze the correlation between them. Receiver operating characteristic (ROC) curve was used to evaluate the efficacy of LVD in predicting lymph node metastasis of early gastric cancer. P<0.05 meant difference had statistical significance.

## RESULTS

The positive expression and LVD of the gastritis group, LGIN group, HGIN group and gastric cancer group gradually increased (Table-I).The positive expression of CD44v6 and LVD in the early gastric carcinoma were correlated with lymph node metastasis and lymphatic infiltration (p < 0.05), but in no correlation with gender, age, tumor site, maximum diameter, degree of tissue differentiation and depth of infiltration (P>0.05, Table-II).The Spearman rank correlation analysis suggested that the positive expression of CD44v6 in the early gastric cancer tissue was in a positive correlation with LVD (r=0.301, P<0.05).The ROC curve analysis suggested that the area under ROC curve of predicting lymphatic metastasis of early gastric cancer based on LVD was 0.837 (95% confidence interval (CI): 0.756~0.91), and the cut-off value was 14; the sensitivity and specificity of LVD in predicting lymphatic metastasis of early gastric cancer were 63.6% and 90.2% respectively.

**Table-I:** Positive expression of CD44v6 and LVD between different groups.

Group	Positive expression of CD44v6 [n(%)]	LVD (n)
Gastritis group (n=60)	2(3.3)	2.65±1.19
LGIN group (n=60)	17(28.3)^a^	4.18±1.45^a^
HGIN group (n=55)	26 (47.3)^ab^	6.36±1.98^ab^
Gastric cancer group (n=100)	56(56.0)^ab^	12.5±4.62^abc^

***Note:***

a indicated P<0.05 comparing to the gastritis group;

b indicated P<0.05 comparing to the LIGN group;

c indicated P<0.05 comparing to the HIGN group.

**Table-II:** Relationships between the positive expression of CD44v6, LVD and the clinicopathological parameters of patients with early gastric cancer in the gastric cancer group.

Clinicopathological parameters		n	Positive expression of CD44v6 [n(%)]	P	LVD (n)	P
Gender	Male	68	36(52.9)	>0.05	12.06±4.20	>0.05
	Female	32	19(59.4)		13.46±5.34	
Age	>60	54	31(57.4)	>0.05	12.31±4.47	>0.05
	≤60	46	25(54.3)		12.75±4.83	
Site of cancer	Cardia and fundus	30	13(43.3)	>0.05	11.02±4.21	>0.05
	Gastric body	28	16(57.1)		13.08±5.40	
	Gastric antrum	42	26(61.9)		13.22±4.18	
Maximum diameter of cancer	>2cm	40	25(62.5)	>0.05	12.81±4.22	>0.05
	≤2cm	60	32(53.3)		12.34±4.90	
Degree of tissue differentiation	High and moderate differentiation	49	25(51.0)	>0.05	11.92±4.17	>0.05
	Low differentiation	51	31(60.8)		13.08±4.99	
Depth of infiltration	Mucosal layer	43	20(46.5)	>0.05	11.68±4.67	>0.05
	Submucosal layer	57	35(61.4)		13.15±4.52	
Lymphatic metastasis	With	37	26(70.3)	<0.05	15.81±4.31	<0.05
	Without	63	30(47.6)		10.59±3.62	
Lymphatic vessel invasion	With	20	19(95.0)	<0.05	17.28±5.12	<0.05
	Without	80	37(46.3)		11.36±3.66	

## DISCUSSION

Gastric cancer is a common digestive tract cancer. The traditional disease pattern of intestinal-type gastric cancer has been widely accepted.^11^ In recent years, the theory of tumor stem cells has opened a new field of vision for the understanding of the origin of tumor. This theory believes that tumor is a stem cell disease,^12^ and malignant tumors originate from very few cancer stem cells with the ability of self renewal and unlimited proliferation. There are two origin theories of gastric cancer stem cells, gastric stem cells and bone marrow derived stem cells. A study has showed that gastric stem cells or bone marrow derived stem cells eventually led to the occurrence of gastric dysplasia or intestinal-type gastric cancer under the effect of gene mutation,^13^ inflammation and injury. In addition, tumor stem cells are also related to tumor invasion and metastasis, which may be the main cause of tumor recurrence and chemoradiotherapy resistance.^14^

CD44 is a multifunctional single-chain transmembrane glycoprotein encoded by single gene, which is highly heterogeneous. It is a highly concerned cancer metastasis related molecule in recent years. CD44V6 is a structural variant of CD44. Currently, it is generally believed that high expression of CD44v6 is related to staging, metastasis and poor prognosis of advanced gastric cancer.^15^ In the precancerous lesion stage or early tumors, the expression of CD44v6 increases. Therefore, some scholars regard CD44v6 as a molecular marker for the diagnosis of precancerous lesions and early tumors, such as pulmonary precancerous lesions and early pancreatic cancer.^16^,^17^ However, few studies have showed the expression of CD44v6 in gastric precancerous lesions or early gastric cancer tissues. The results of this study showed that the positive expression of CD44v6 in the gastritis group, LGIN group, HGIN group and gastric cancer group gradually increased, which were basically consistent with the results of Da et al.^18^ It indicated that CD44v6 played an important role in the early malignant transformation of gastric mucosa, which will be helpful for the diagnosis of precancerous lesions and early gastric cancer. There was no significant difference in the positive expression of CD44v6 in the gastric cancer group and the HGIN group. It might be because those high-grade gastric intraepithelial neoplasias were similar to cancer tissue in the perspective of histomorphology. It was also found that the positive expression of CD44v6 was related to lymph node metastasis and lymphatic invasion in early gastric cancer, which indicated that CD44v6 might be involved in the lymph node metastasis of early gastric cancer. The reason might be that CD44v6 as a stem cell factor had lymphoid disguise in the process of tumor cell growth and entered the lymph nodes while escaping from the identification and killing of the immune system, which is also mentioned in a study of Hofmann et al..^19^ but the specific machine has not been clarified and remains to be further studied.

LVD refers to the number of lymphatics per unit area. Early studies suggest that tumor lymph node metastasis is related to tumor invasion in peripheral lymphatic vessels. The increase of LVD is associated with lymphangiogenesis. Tumor cells transfer to local lymph nodes through lymphatic formation and lymphatic infiltration; therefore, LVD, lymphatic infiltration and lymph node metastasis are closely correlated. Studies suggested that LVD could be used as an indicator for predicting lymph node metastasis in lung cancer and colon cancer.^20^,^21^ The results of this study showed that LVD in the gastritis, LGIN, HGIN and gastric cancer groups increased gradually. The further study found that LVD was associated with lymph node metastasis and lymphatic infiltration, which revealed that the increase of LVD might be one of the pathological features of lymph node metastasis in early gastric cancer. Lee et al. considered that LVD was an independent predictor of lymph node metastasis in early gastric cancer and lymph node metastasis could be predicted based on LVD.^22^ The results of this study showed that the area under the ROC curve of predicting lymph node metastasis of early gastric cancer was 0.837 (95% CI: 0.756~0.910) of lymph node metastasis in early gastric cancer, and the cut-off value was 14; the sensitivity and specificity of predicting lymph node metastasis was 63.6% and 90.2% respectively. All the findings demonstrated that LVD had high values in predicting lymph node metastasis in early gastric cancer. In addition, this study also found that the positive expression of CD44v6 was positively correlated with LVD in early gastric cancer. It was concluded that the detection of CD44v6 expression and LVD could use for predicting lymph node metastasis in early gastric cancer.

### Limitations of the study

But the sample size of this study was small, and the samples came from single center, which might lead to bias. Deep studies with larger sample size and multiple centers are needed to clarify the important role of CD44v6 and LVD in gastric cancer.

## CONCLUSION

The expression of CD44v6 and LVD will increase in early gastric cancer tissues, and they are related to lymph node metastasis. The detection of CD44v6 expression and LVD can be used for predicting the risk of lymph node metastasis in gastric cancer patients and guide clinical treatment, prognosis evaluation and selection of treatment schemes.

### Authors’ Contribution

**YTS & XWY:** Study design, data collection and analysis.

**YTS, YXW & MDL:** Manuscript preparation and revising.

**YTS & ZHZ:** Review and final approval of manuscript.
